# Birth of a wild black howler monkey (*Alouatta pigra*) at an anthropogenic site

**DOI:** 10.1007/s10329-022-01022-z

**Published:** 2022-11-05

**Authors:** Anaid Cárdenas-Navarrete, Sarie Van Belle

**Affiliations:** 1 Department of Integrative Biology, University of California, Berkeley, CA USA; 2 Posgrado en Ciencias Biológicas, Universidad Nacional Autónoma de México, Mexico City, Mexico; 3 Instituto de Biología, Universidad Nacional Autónoma de México, A.P. 70-153, 04510 8 Mexico City, Mexico; 4 Department of Anthropology, University of Texas at Austin, Austin, TX USA

**Keywords:** Arboreal primate, Neotropics, Offspring, Parturition

## Abstract

We present the first description of a diurnal live birth of a wild black howler monkey (*Alouatta pigra*). The mother formed part of a group of five individuals inhabiting an anthropogenic setting in the tropical lowlands of southeast Mexico. A total of 7 h and 50 min passed from the rupture of the amniotic sac early in the day to the crowning of the infant from the birth canal. The delivery of the infant lasted ~ 3 min. We describe the event while referencing images and time points in a supplementary video recording. We place our findings in the context of the available reports of live births in the *Alouatta* genus, time of day, birth duration, and group activity budget on the day of the birth. While primates tend to give birth at night to reduce complications from group interactions, the observed birth took place during the day, which may have been possible due to an alteration in group time allocation. Our report provides in-depth details of the events of a birth and important information regarding the natural history of the black howler monkey.

**Supplementary Information:**

The online version contains supplementary material available at 10.1007/s10329-022-01022-z.

## Introduction

Reproductive events such as live births in wild primates are rare to witness due to a variety of factors, including the challenges of observing arboreal primates, the seclusive nature of most primate species, and the rarity of wild primate births stemming from their relatively long gestation periods and interbirth intervals (Strier 2001; Van Belle and Bicca-Marques 2015). Howler monkeys (genus *Alouatta*) are an example of primates with these factors, including an estimated gestation period ranging from 152 to 195 days and an interbirth interval of 16–23 months (Dias et al. 2011; Van Belle et al. 2009; Van Belle and Bicca-Marques 2015), leading to difficulties observing birth events in the wild. Given that howler monkeys’ behavioral flexibility permits them to survive in degraded habitats (Arroyo-Rodríguez and Dias 2010; Bicca-Marques 2003), it is important to report instances of live births in these contexts and reflect, if possible, on the potential direct and indirect impacts of habitat degradation on crucial reproductive events (Cañadas Santiago et al. 2020; Cristóbal Azkarate et al. 2017; Martínez-Mota et al. 2007).

Here, we present a detailed description, accompanied by video recordings, of a live birth of a male black howler monkey (*Alouatta pigra*) in an anthropogenic setting in Chiapas, Mexico. We place our findings in context with available reports of live births from the *Alouatta* genus (Table 1), including *A. palliata* (Dias 2005; Moreno et al. 1991; Nisbett and Glander 1996), *A. caraya* (Kowalewski and Zunino 2004; Peker et al. 2009; Rumiz 1990), *A. guariba clamitans* (Martins et al. 2015), and *A. arctoidea* (Sekulic 1982). We also provide novel information on four other black howler monkey births from the nearby protected forest, Palenque National Park (Table 1). We describe the main birth event from the amniotic sac rupture to the delivery of the infant, and we compare the activity budgets of the group on the day of the birth with their average activity budget throughout the observation period. Our report provides a rare and important look into the birthing process of a wild black howler monkey living in an anthropogenic setting.

**Table 1:** Summary of reported births in the genus *Alouatta*

**Species**	**Location**	**Forest type**^**a**^	**Date (dd/mm/yy)**	**Time of birth**^**b**^	**Mother’s birthing history**^**c**^	**Mother’s behavior**	**Infant’s sex**	**Proximity to group members**	**Group composition before birth **^**d**^	**Reference**
*A. arctoidea*	Guarico, Venezuela	SA	1/10/1979	15:35	MP	Squatting posture / Chewed umbilical cord / Fed on placenta	M	Close to AM and JF only	3 AF, 2 AM, 1 SAM, 1 JF, 2 I	Sekulic (1982)
		SA	29/03/80	15:50		Vulvar contractions / Chewed umbilical cord	U	5 m	1 AM, 1 AF, 2 I	Sekulic (1982)
*A. caraya*	Northern Argentina	CF	27/07/06	17:45	MP	Touched genitals / Squatting posture / Fed on placenta	U	17 m	1 AM, 2 AF, 1 JM	Peker et al. (2009)
		CF	20/09/07	16:46	MP	Touched genitals / Squatting posture / Assisted birth with hand / Fed on placenta	U	40 m	2 AM, 4 AF, 2 SAM, 1 SAF, 1 JF, 1 I	
		FF	23/09/06	18:52	MP	Touched genitals / Squatting posture / Licked hands / Fed on placenta	U	6 m	1 AM, 1 AF, 1 JM	
		FF	24/09/07	11:59	MP	Touched genitals / Squatting posture / Urinated / Assisted birth with both hands / Fed on placenta and umbilical cord	U	12 m	1 AM, 1 AF, 2 JM	
		CF	24-25/09/04	Night	PP	-	F	-	3 AM, 3 AF, 1 SAF, 1 IM, IF	
		CF	11-12/05/06	Night	MP	-	U	-	1 AM, 3 AF, 1 JM, 1 JF, 1 IF	
		CF	20-21/09/06	Night	MP	-	U	-	2 AM, 3 AF, 1 JM, 1 JF, 2 IF	
*A. guariba clamitans*	Porto Alegre, Brazil	FF	27/10/13	14:33	MP	Fed on placenta and umbilical cord / Licked infant's body	M	2 m	1 AM, 2 AF, 1 SAM, 1 SAF, 3 JM, IF	Martins et al. (2015)
*A. palliata*	Guanacaste, Costa Rica.	GF	20/12/91	15:08	PP	Fist clenching / Restless behavior / Bit tail / Rubbed and pulled genitals / Squatting posture / Defecated and urinated	U	-	2 AM, 3 AF, 1JM	Nisbett and Glander (1996)
	Veracruz, Mexico	IS	6/1/1999	14:08	MP	Restless behavior / Squatting posture / Touched genitals / Licked-smelled hand / Short vocalizations / Fed on placenta	U	15 m	13 MA, 21 FA, 10 SA, 9 J, 6 I	Dias (2005)
*A. pigra*	Chiapas, Mexico	FF	18/05/19	17:44	MP	Restless behavior and walking / Squatting posture	M	< 5 m	1 AM, 2 AF, 1 JM, 1 IM	Present report
		CF	8/11/2006	15:04	MP	Restless behavior / Squatting posture / Assisted birth with hand / Fed on placenta	U	-	1 AM, 2 AF	
		CF	20/02/10	Midday	MP	Not observed / Fed on placenta	F	> 30 m	3 AM, 1 SAM, 2 AF, 1 JM, 1 IM	
		CF	19/07/15	Night or dawn	PP	Not observed / Found placenta on ground (Fig. 2)	F	-	1 AM, 2 AF	
		CF	22/05/18	15:22	MP	Squatting posture	M	< 15 m	2 AM, 4 AF, 2 JF, 2 JM, 2 IF	

^a^*CF* Continuous Forest, *FF* Forest Fragment, *GF* Gallery Forest, *IS* Island, *SA* Savana

^b^Ten of the studies reported data on the duration of birth from crowning to complete emergence ranging from <1 to 7 min total

^c^*PP* Primiparous, *MP* Multiparous

^d^*M* male, *F* female, *A* adult, *SA* subadult, *J* juvenile, *I* infant, *U* unknown

## Methods

### Study site

The birthing event took place in a 13-ha forest fragment (17°30′N, 91°59′W) located in the anthropogenic landscape outside of Palenque National Park (PNP) in Chiapas, Mexico. Annual temperature in Palenque ranges from 20 to 32 °C (CONAGUA 2010). Mean annual rainfall in the area is 2079 mm divided into two seasons of higher rainfall from June–December (monthly mean, 215 ± 65.9 mm) relative to January–May (monthly mean, 94 ± 38.3 mm; (CONAGUA 2010). The Palenque area, which originally consisted of homogeneous lowland tropical rainforest, consists of an urban center, the protected forest of PNP (UNEP-WCMC 2022) that surrounds the ancient Maya ruins of Palenque, and a heterogeneous landscape in between (Klass et al. 2020; Levey et al. 2021). The forest fragment where we observed the birth primarily consisted of a 30-year-old rubber tree (*Hevea brasiliensis*) plantation (~ 11 ha) and the grounds of a hotel (~ 2 ha). Native (e.g., *Inga* sp., *Bursera simaruba*, and *Zanthoxylum riedelianum*) and non-native (e.g., *Ficus benjamina* and *Mangifera indica*) vegetation within and around the borders of the plantation served as food resources for the black howler monkey group. A vegetation survey based on ten 50-m transects (0.1 ha) revealed that the average diameter at breast height (DBH) in the fragment was 37.1 ± 26.5, while the tree height was 13.6 ± 6 m (*N* = 50; Cárdenas-Navarrete 2020). The forest fragment was inhabited solely by the study group.

### Data collection

We registered the birth while conducting a study regarding the influences of vegetation structure present in degraded habitats on the patterns of movement, diet, and activity of black howler monkeys (Cárdenas-Navarrete 2020). Before the birth, the group inhabiting the site consisted of an adult male, two adult females, one juvenile male (offspring of the birthing female), and one infant male. We observed this group for 17 days in May and 24 days in October of 2019. The individuals of the group were habituated to human presence since they did not display avoidance behavior to our observation team and generally ignored our presence. This is likely due to a previous observational study of the group (Klass et al. 2020) and constant human presence within the site. After locating the focal group in the mornings, we collected instantaneous scan samples every 15 min to register the activity of each individual, as well as the geographic location of the group with a GPS device. Behavioral categories included: (1) feeding (eating plant material), (2) resting (lack of movement or participation in any notable social activity), (3) traveling (movement, usually involving all group members, beyond the confines of a single tree), (4) moving (movement within a single tree crown or branch), (5) social interactions (such as play or sexual interactions), (6) vocalizing (including howling and grunting), and (7) other (any other behavior not covered by the six previous categories). We collected extraordinary events (e.g., individuals traveling by ground, aggressive encounters, birth events, etc.) ad libitum along with time, location, and identity of the individuals involved.

We observed and video recorded a live birth of an infant male black howler monkey on May 18, 2019. From a distance of ~ 20 m and a camera with zoom (Nikon P900 – 83 × wide optical zoom), we began recording photos and videos of the major events during the birth. Since we stopped recording every time the mother went out of sight, the supplementary video contains short recordings compiled in a continuous series. We reference specific moments in the footage with descriptions and the actual hour of the day for each major event of the birth process. Therefore, the references to time durations in the supplementary video do not reflect the actual duration of the birth. Human presence other than the observers was common on the hotel grounds, and one onlooker can be heard on the video recording clapping and exclaiming during the birth of the infant.

## Results

### Event description

**Fig. 1:**
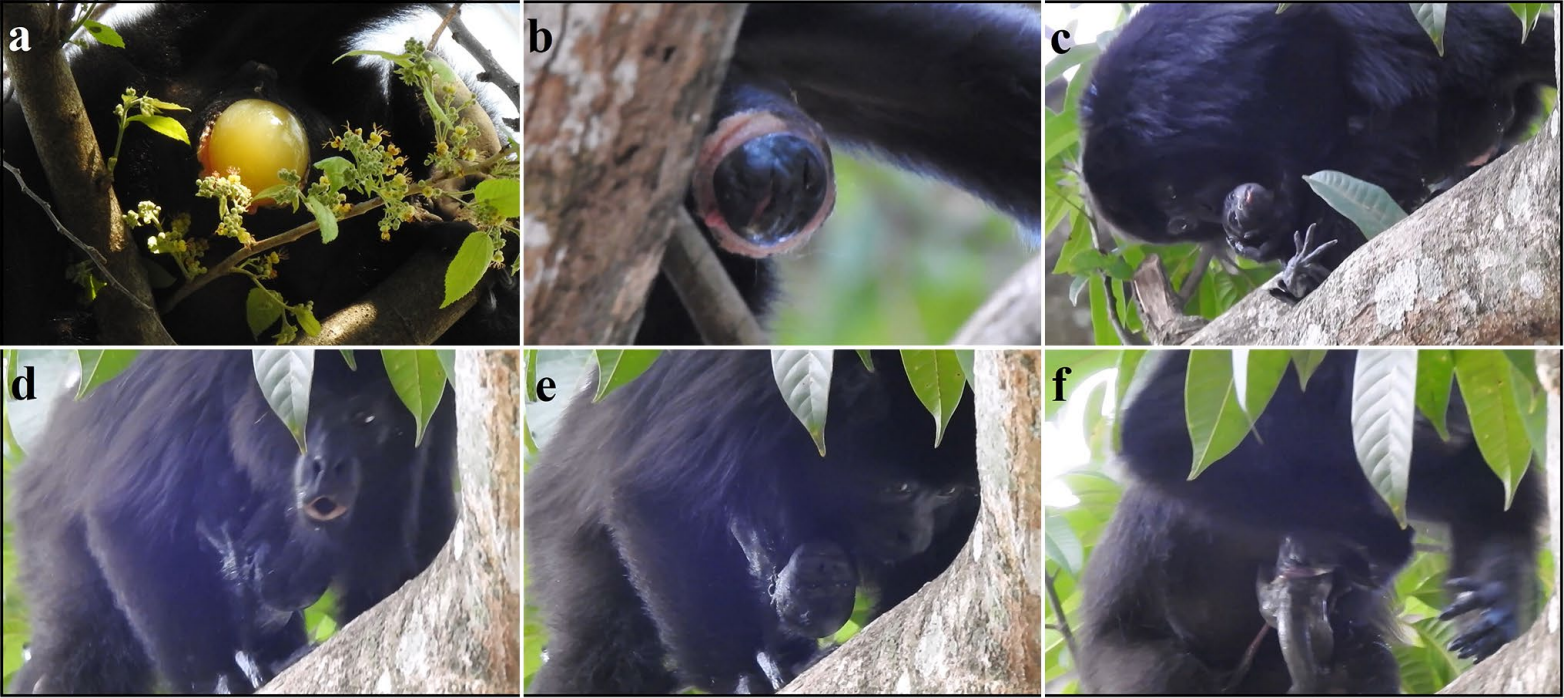
Still photo series from video recordings of the live black howler monkey birth in Palenque, Chiapas, Mexico. **a** Genitals of the mother before the rupture of the amniotic sac; **b** infant howler monkey as it emerges from the birth canal; **c** mother holding the infant while inspecting him, the infant’s hands grasping his mother, and the open eyes of the infant; **d** mother participating in a group howl with the infant hanging from her chest; **e** mother and the infant clinging to the mother’s chest with his eyes open; **f** umbilical cord around the infant’s body while still connected to the mother. Photo credit: ACN

We found the mother, a multiparous female, with her group on the morning (09:27 h) of May 18, 2019. Upon locating the group, we noticed a yellow and glossy amniotic sac coming out of the genitals of the mother (Fig. 1a). At 9:54 h, the amniotic sac ruptured, and a large volume of liquid was released. Throughout the rest of the day and leading up to the birth, fluid would occasionally fall from the mother’s protruding genitals. From 12:05 to 13:00 h, the individuals of the group huddled (rested in close contact) with the mother, making observation of the mother difficult. This group behavior, which included the adult male, was a rare occurrence during our observation of the group. We noted that the mother seemed restless at times, making small movements in the tree while the other group members rested.

Ten minutes before the beginning of the birth (17:34 h), the group huddled close to the mother again, 12 m high in a mango tree (*M. indica*) with exposed lower branches. At 17:44 h, 7 h and 50 min after the amniotic sac ruptured, the mother started pacing on the tree branches, and we saw for the first time the head of the infant crowning (Fig. 1b; at 0:02 s of supplementary video). The mother stood in a squatting posture while assisting the delivery of the semi-exposed infant (from 0:19 to 0:31 s of video) until the infant fully emerged at 17:47 h. The mother grasped the fully born infant and held his head in her hands (at 0:32 s of video). The total time between the crowning and the delivery of the infant was ~ 3 min. Once out, the infant held on fast to his mother, displaying immediately a strong grip (Fig. 1c; at 0:34 s of video). The mother inspected her infant and held him close to her chest while looking at a human onlooker not related to the study. The human presence other than the observers was an unintended consequence of our presence at the site. The eyes of the infant opened immediately after the birth (noticeable at 0:42 s of the video), and the umbilical cord was around the torso of the infant (from 0:42 to 0:53 s of the video). Seconds after the infant’s birth, the adult male started howling and accompanied by both adult females (at 0:56 s of video). The cause of the group howl was unknown. The infant remained clutched to the chest of the mother throughout the howling bout (Fig. 1d; at 0:56 s of video). The umbilical cord remained attached to the mother and the infant throughout the observation (Fig. 1e–f; at 1:37 s of video). From 1:49 to 1:51 of the video and about 3 min after being born, the infant made suckling mouth movements and turned his head towards the breast of the mother. A neighboring group also started howling after the focal group had finished calling (from 1:49 to 2:01 s of video). We left the group for the day at 18:05.

Throughout the observation day, the adults of the group rested for 85.4% of their time, fed for 4.9%, moved for 2.9%, vocalized for 1.9%, had social interactions for 2.9%, and traveled between trees for 1% of their time. During the total study period between 9:30 to 18:00 h (observation time on the day of the event), they rested for 70% of the time, fed for 16.6%, moved for 2.3%, vocalized for 1%, had social interactions for 3.6%, and traveled for 6%. Their travel distance on the day of the birth was 41 m.

**Fig. 2:**
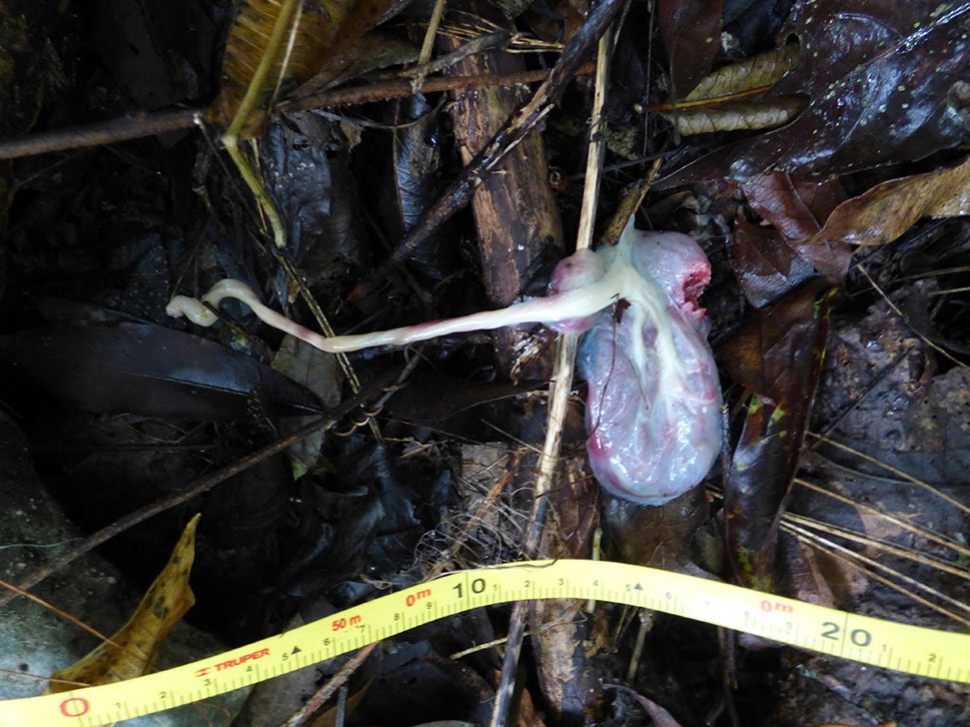
Photograph of placenta of an *Alouatta pigra* female, found after a night birth at Palenque National Park on July 19, 2015. Photo credit: SVB

We made notes during the following observation day on May 19, including a completely severed umbilical cord and much less moisture present on the infant and mother. We did not witness the mother consuming the placenta or find it on the ground the next day (see Fig. 2 for an example of a black howler monkey placenta from PNP). There were instances of infant examination by the other female of the group and the juvenile son of the mother. Also of note was a group travel where the group traveled on the ground due to a lack of connection between the tree crowns. The mother seemed hesitant to travel with the group, waiting for 3 min before descending to the ground with her infant positioned on her abdomen. We noticed that the infant was nursing throughout the next day of observation. The infant was present throughout the remainder of the study (end date November 2, 2019).

## Discussion

We present and describe a video recording of a live black howler monkey birth in an anthropogenic setting in Palenque, Chiapas, Mexico. This observation represents the first report of live birth of a wild black howler monkey, adding valuable information and details to the natural history of this species living in disturbed habitats.

Reports of wild howler monkeys giving birth often lack information regarding the pre-partum phase of the event, likely due to the near-instantaneous period between amniotic sac rupture and birth in most documented cases (Dias 2005; Martins et al. 2015; Nisbett and Glander 1996; Sekulic 1982). Due to a lengthy period between birth stages on the day of the event, we report details on the group’s activity budget from before the rupture of the amniotic sac to the crowning of the infant from the birth canal. The 7-h 50 min period appears long compared to other reported births in *Alouatta* (< 1–3 min; Table 1). Empirical examples to explain the wide deviation from our observation to the other *Alouatta* births are lacking, but theoretically, it is possible that the mother underwent a form of reproductive suppression at the parturition stage, either from a stress response to environmental conditions or an energy intake imbalance (i.e., greater energy output than input; Beehner and Lu 2013). It is also possible that the time between amniotic sac rupture and crowning of the infant is a normal range of variation within the species, but due to the rarity of observing these events in the wild, it has not been previously reported. The 3-min duration from crowning to complete emergence of the infant in our observation is similar to other reports of live births in howler monkeys (< 1–7 min; Table 1).

Even though primates tend to give birth at night, daytime births have been registered in both experienced and non-experienced females (Dias 2005; Martins et al. 2015; Nguyen et al. 2017; Nisbett and Glander 1996; Peker et al. 2009; Sekulic 1982). While giving birth at night may reduce the likelihood of intragroup interactions with the mother and increase infant survival by taking place when the group is resting (Nguyen et al. 2017; Sekulic 1982), diurnal births may not be strongly selected against in some primate species (Nguyen et al. 2017). This is also supported by observations of four live births of black howler monkeys in nearby PNP, including three diurnal births between 11:40 and 17:40 h (Table 1). The altered group time allocation during the day of the event may be a strategy to reduce energy expenditure and group travel, which may decrease complications for the mother (Sekulic 1982). The 41-m group travel distance on the day of the birth differed substantially from the daily average distance of 316 ± 150 m during the study period (Cárdenas-Navarrete 2020). Additionally, the mother was not wary of the other group members, as shown by the close huddling behavior noted before the birth. Another hypothesis that may reduce selection pressure against diurnal births in black howler monkeys includes anti-predator vigilance behavior from the rest of the group during the day, which would provide an advantage for the vulnerable mother and infant (Turner et al. 2010). Anti-predator vigilance from the group may include huddling around the mother or loud calling immediately after giving birth, as we observed.

Our video recording of a birth of a male infant black howler monkey offers a rare glimpse into the timing and process of the live birth of an elusive primate species, including the time from the rupture of the amniotic sac to the infant leaving the birth canal and social group dynamics during the day of birth. Our findings are an important addition to the birth tendencies of howler monkeys and provide a novel record for the black howler monkey. Our record, combined with other births in the study area, also provide information for theoretical questions on when and how wild primates give birth.

## Supplementary Information

Below is the link to the electronic supplementary material.

Supplementary file1 (MP4 255540 KB)
